# Navigating the manufacturing, testing and regulatory complexities of regulatory T cells for adoptive cell therapy

**DOI:** 10.3389/fimmu.2025.1626085

**Published:** 2025-07-16

**Authors:** Larissa A. Pikor, Sindhu Arivazhagan, Michael Mendicino, Sarmitha Sathiamoorthy

**Affiliations:** ^1^ AspireBio Consulting, Toronto, ON, Canada; ^2^ Hybrid Concepts International LLC, Grand Island, NY, United States

**Keywords:** Treg, ACT, CMC, analytics, manufacturing, regulatory

## Abstract

Regulatory T cells (Tregs) are a small, unique subset of suppressive T cells that play a pivotal role in regulating the immune system by maintaining tolerance to self-antigens and preventing autoimmune disease. Adoptive transfer of Tregs for the treatment of autoimmune disorders such as arthritis and allergic airway inflammation, graft-*versus*-host disease (GvHD) and rejection following transplant have shown promise in early phase clinical trials. Despite over a decade of clinical manufacturing, there remains significant manufacturing and testing complexities for this class of therapies, including the need for specialized facilities and highly trained personnel that make clinical and commercial supply challenging. In this review, we discuss the current Chemistry, Manufacturing and Controls (CMC) and regulatory complexities and challenges to the development and commercialization of Treg therapies. Some of these are specific to Tregs while others are broadly applicable to the field of cell-based therapy. Discussion topics include the importance of starting material selection, the availability of GMP quality reagents and material, isolation and characterization of regulatory T cells, cGMP manufacturing considerations and limitations, the complexity of testing, release and distribution of cell-based therapies, as well as the regulatory challenges associated with Treg therapy. Treg cell therapies can be fraught with technical challenges which are mirrored by a sponsor’s ability to meet regulatory requirements. Despite these hurdles, the promise of Tregs as a therapeutic for the treatment of autoimmune and other diseases warrants continued development.

## Introduction

Conventional regulatory T cells (Tregs) are a subpopulation of CD4^+^ T cells characterized by the expression of the high-affinity IL-2 receptor alpha chain (CD25), transcription factor forkhead box P3 (Foxp3) and low expression of the IL-7 receptor alpha chain (CD127), (CD4^+^CD25^+^Foxp3^+^CD127^lo/-^) that are essential in regulating other cells in the immune system, to maintain immune homeostasis, prevent autoimmunity and limit chronic inflammatory diseases (1, 2). While non CD4+Foxp3+ Tregs have been identified (CD4^-^CD8^+^, CD4^-^CD8^-^ and CD4^+^Foxp3^-^(Tr1)) (3), non-conventional Tregs are outside the scope of this review. Tregs are divided into two main subsets; naturally occurring Tregs derived from the thymus (also referred to as thymic Tregs (tTregs) and present in cord blood) and peripherally induced Tregs (pTreg) derived from conventional CD4^+^ cells in secondary lymphoid organs that encounter antigen in the presence of TGF-B (4). Despite differences in origin, there are currently no known markers to distinguish between human tTregs and pTregs and both subsets function to suppress immune responses towards both self and non-self-antigens by inhibiting the activation, proliferation and cytokine production of CD4^+^ and CD8^+^ T cells through multiple mechanisms (5). These include; the production of anti-inflammatory/inhibitory cytokines such as transforming growth factor beta (TGF-B) and IL-10, metabolic disruption (production of adenosine) and cytokine starvation (sequestering IL-2 via CD25) both of which limit effector T cell function and expansion, as well as modulation of antigen presenting cell (APC) maturation and/or function through the expression of inhibitory cell surface receptors such as cytotoxic T cell-associated antigen (CTLA-4), lymphocyte activation gene 3 (LAG 3) and T cell immune receptor with Ig and ITIM domains (TIGIT) (4–7). Many of the suppressive activities of Tregs function in an antigen independent manner (dominant bystander suppression) enabling Tregs to suppress effector cells of diverse specificities (8).

The multi-faceted activity of Tregs, and their ability to exert their function in both lymphoid and non-lymphoid tissues (i.e., at the site of inflammation) afford a unique therapeutic opportunity to treat the complex pathophysiology of autoimmune and inflammatory diseases. To date, more than 50 clinical trials (ongoing or completed) have been conducted using Tregs (www.clinicaltrials.gov). The search was conducted by entering the term “Treg infusion” into the intervention/treatment search and focusing on interventional trials. The most common indications include graft *versus* host disease (GvHD), organ transplantation and autoimmune diseases such as Chron’s, multiple sclerosis, type I diabetes (TID) and lupus (9, 10). These have all been early phase trials (Phase 1/2) investigating the safety and biological activity of Tregs, with the majority sponsored by academic and medical institutions. Trial results have produced clear evidence of the feasibility and safety of Treg therapy in autoimmunity and transplantation, but early efficacy data has been limited. Randomized, controlled Phase 3 studies have yet to be published, however later stage industry sponsored trials are underway with Rapa Therapeutics launching a Phase 2b study of an autologous Treg/Th2 hybrid T cell product in amyotrophic lateral sclerosis at the end of 2024 that is currently recruiting (NCT04220190).

Five key decisions are made at the preclinical/candidate selection stage of product development that dramatically impact the feasibility of manufacturing, testing and release of clinical lots of Treg products to ensure that they meet quality and compliance expectations by the regulator. These include: 1) the source of material used for isolation of Tregs, 2) whether the product is for autologous or allogeneic use, 3) if the product is polyclonal or antigen specific (i.e., genetically modified or not - TCR or CAR-T Tregs) 4) what subpopulation of Tregs are desired (i.e.: what cell surface markers will be used to isolate Tregs) and 5) whether the product will be cryopreserved or administered fresh. Awareness of the complexities associated with each of these decisions early during product development can help facilitate and expedite clinical development and should be performed in collaboration with CMC and regulatory experts. In this review, we summarize current practices in Treg manufacturing and discuss the CMC and regulatory considerations and complexities associated with each process step.

## Manufacturing of Treg therapies

**Figure 1:**
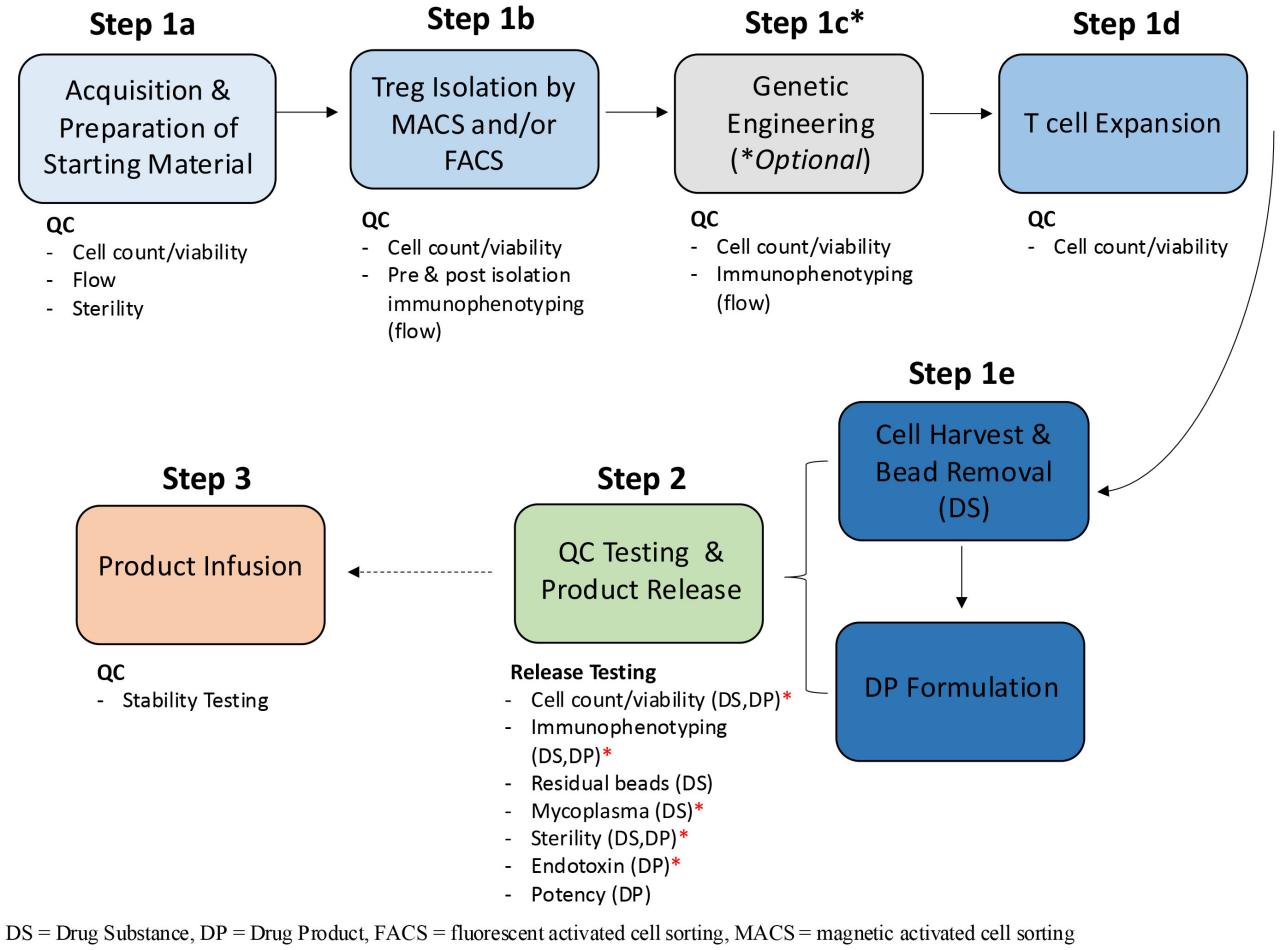
Treg manufacturing process flow diagram. The clinical manufacturing process for Treg therapies is broadly categorized into three phases: manufacturing, Quality Control (QC) testing and product release and product infusion and monitoring. The manufacturing phase can be further subdivided into multiple unit operations which include Treg isolation, genetic engineering, cell expansion and cell harvest. The quality (viability) and quantity (cell count and or immunophenotyping by flow cytometry) of Tregs are quantified at each stage of the manufacturing process; upon receipt of the starting material (Step 1a), pre and post isolation (1b), post engineering (1c) if performed, throughout expansion (i.e. re-seeding, addition of CD3/CD28 stim) 1d) and at harvest of the DS (1e). Once formulated, the DP undergoes release testing (Step 2). In some instances, product infusion may occur before all release tests are completed, so long as a minimal set of conditional release assays have been performed. This is especially true for autologous products and is represented by the hashed arrow between step 2 and 3. The minimal set of assays required for release (minimum release testing panel), before the product can be infused into the patient, are annotated by red asterisks and include; cell count, phenotype, endotoxin, mycoplasma by PCR and gram staining and/or BacTALERT.

The clinical manufacturing process for Treg therapies involves manufacturing, testing, release and stability (**Figure 1**). Manufacturing is a complex and labor-intensive process comprised of five stages (regardless of the source of the starting material or nature of the therapy (autologous vs. allogeneic)); starting material acquisition, isolation of Tregs, *ex vivo* expansion, cell harvest and formulation. Several different clinical manufacturing methods have been published, with variations in virtually all key process parameters, including markers used to isolate Tregs, media, activation reagents, IL-2 concentration, restimulation timing, culture duration and formulation (11). The lack of systemic studies to define critical process parameters and optimal reagents, has resulted in uncertainty regarding the best methods to isolate, expand and formulate Tregs for clinical use.

There are currently three types of Treg products being developed for clinical use; autologous Tregs (non-gene-modified or edited), allogeneic non-modified Tregs and allogenic engineered (gene modified or edited) Tregs. Autologous Tregs are the most prevalent product as peripheral blood is easily accessible and there is no risk of rejection following infusion. Unmodified allogeneic Tregs have been used in immunocompromised patients to treat GvHD, however even in these patients, these cells are quickly recognized and destroyed by the host immune system, resulting in short lived therapeutic efficacy (12, 13). Engineered allogenic Tregs are modified by non-viral gene modification/editing (CRISPR-Cas) or viral gene modification/editing to express a synthetic chimeric antigen receptor (CAR) or T cell receptor (TCR) that recognizes a target antigen of interest, making these cells antigen-specific rather than polyclonal. Additional genetic engineering strategies such as knockout of Beta-2 microglobulin and/or CIIT2 to eliminate class I and II human leukocyte antigen (HLA) molecules respectively, and prevent rejection of polyclonal allogeneic Tregs are being assessed preclinically (14, 15). As these modifications could lead to increased natural killer (NK) cell-mediated elimination, strategies to express non-classical HLA molecules (HLA-E or CD47) are also being developed (16, 17). The manufacturing, testing and regulatory implications associated with each class of Tregs are summarized in **Table 1**.

**Table 1:** Manufacturing and regulatory considerations for different Treg cell products.

Process Step		Autologous, Polyclonal Unmodified Tregs	Allogeneic, Polyclonal Unmodified Tregs	Allogeneic, Gene Modified and/or Edited Tregs
1a	Starting Material	PBMC are the most easily and readily accessible source of Tregs With personalized therapy, there is no risk of rejection Frequency of Tregs is variable. Disease state can further reduce Treg numbers and impair their function.	High proportion of naive cells UCB has low total numbers of Tregs (100x less than thymus) Allogenic products require partial HLA matching and extensive screening to ensure safety	
1b	Isolation	Both FACS and MACS are routinely used The inability of MACS to discriminate expression level (i.e.: low vs. positive vs. high) leads to reduced purity. FACS can use multiple markers and discriminate expression levels to select a specific, pure population. It is a more labor intensive and time-consuming process that can impact yield.	MACS predominantly used Thymus: Lack of conventional T cells, means that CD25+ marks Tregs. One-step isolation protocol can be used to obtain a pure population of Tregs (See Treg Isolation section)	
1c	Genetic Engineering	N/A	N/A	Additional material requirements, and increased testing Decision of when to perform editing (before or after expansion) will impact yield and purity. Longer, more complex manufacturing process that requires additional specialized equipment for transduction or electroporation Editing can negatively impact viability and total cell number/yield
1d	*Ex vivo* Expansion	Expansion of autologous products is highly variable, higher risk of manufacturing failures. Variability in product yield and phenotype at end of manufacturing Higher manufacturing costs	Tregs are from healthy donors, expansion is more reproducible Longer expansion step relative to autologous therapies due to limited starting cell number (UCB) May require additional culture additives (rapamycin) to ensure purity if 1-step MACS performed	Longer expansion phase required due to negative impact of editing on cell viability
1e	Formulation	Cryopreservation allows for flexibility around dosing, reduces logistical complexities and the need for expedited release testing, however, it is known to negatively impact cell viability, marker expression and expansion/survival post infusion. Freshly infused Tregs have greater viability, but need to be manufactured at or close to the clinical site (de-centralized) and will have a short shelf-life		
2	Release Testing	Establishing appropriate CQAs and specifications can be difficult due to variability in product	Additional safety testing	Production and testing of editing tools are time consuming and costly Additional testing requirements to demonstrate product stability & safety (i.e., identification of on and off-target editing)
3	Product Infusion	Non-specific immune suppression disease stabilization	Non-specific immune suppression Can enable rapid dosing for acute indications (i.e., ARDS, stroke) with the potential for repeat dosing Rapid clearance following infusion (even in immunosuppressed patients)	Enhanced potency relative to polyclonal Tregs may allow for transfer of fewer cells Engineering can enhance specificity, functionality, persistence and ability to traffic to the site of inflammation

CQA, critical quality attribute; FACS, fluorescent activated cell sorting; HLA, human leukocyte antigen; MACS, magnetic-activated cell sorting; PBMC, peripheral blood mononuclear cells; UCB, umbilical cord blood.

The manufacturing of cell-based therapies must adhere to cGMP guidelines, requiring cGMP compliant reagents and is subject to multiple regulatory (FDA & IHC) guidance, the most pertinent of which are summarized in **Table 2** (the complete list can be found in SI1). As living drugs, control of reagents, starting material, the manufacturing process and the final product is essential to ensure product safety and consistency. Sourcing cGMP compliant materials and reagents is not always possible, and when available, suppliers are often limited, leading to potential supply chain issues. Acquiring the necessary cGMP compliant reagents and materials, having experienced personnel, qualified equipment and facilities remains a significant barrier to Treg manufacturing. These individual challenges will be discussed throughout this review.

**Table 2:** Regulatory guidance relevant to Treg therapies.

Regulatory Body	Guidance	Draft (Y/N)	Year	Application
FDA	Recommendations for Determining Eligibility of Donors of Human Cells, Tissues, and Cellular and Tissue-Based Products (HCT/Ps)	Y	Jan 2025	Applies to screening of donors of human cells or cell–based products such as Tregs
FDA	Frequently Asked Questions — Developing Potential Cellular and Gene Therapy Products	Y	Nov 2024	Provides recommendations for interactions with FDA and issues that may arise during Treg product development and characterization, design of nonclinical studies and design of human trials
FDA	Considerations for the Use of Human-and Animal-Derived Materials in the Manufacture of Cell and Gene Therapy and Tissue-Engineered Medical Products	Y	April 2024	Applies to human and/or animal origin materials such as reagents, feeders, excipients used in the Treg manufacturing process or those used to manufacture starting materials used in the process
FDA	Safety Testing of Human Allogeneic Cells Expanded for Use in Cell-Based Medical Products	Y	April 2024	Applies to allogeneic cells of human origin if they are used to manufacture Tregs
FDA	Considerations for the Development of Chimeric Antigen Receptor (CAR) T Cell Products	N	Jan 2024	Provides specific CMC, non-clinical and clinical recommendations for CAR Tregs
FDA	Human Gene Therapy Products Incorporating Human Genome Editing	N	Jan 2024	Applies if Tregs were gene edited *ex vivo*
FDA	Potency Assurance for Cellular and Gene Therapy Products	Y	Dec 2023	Provides recommendations for developing a science- and risk-based potency assurance strategy for Tregs
FDA	Manufacturing Changes and Comparability for Human Cellular and Gene Therapy Products	Y	Jul 2023	Applies to management of manufacturing process changes for Tregs and recommendations for comparability studies
FDA	Long Term Follow-Up After Administration of Human Gene Therapy Products	N	Jan 2020	Applies if Tregs were genetically modified *ex vivo*
FDA	Source Animal, Product, Preclinical, and Clinical Issues Concerning the Use of Xenotransplantation Products in Humans	N	Dec 2016	Applies if the Tregs are a xenotransplantation product
FDA	Recommendations for Microbial Vectors Used for Gene Therapy	N	Sep 2016	Applies if Tregs are genetically modified or gene edited *ex vivo* using microbial vectors
FDA	Determining Donor Eligibility for Autologous Donors of Blood and Blood Components Intended Solely for Autologous Use – Compliance Policy	N	Aug 2016	Applies to screening of donors of human cells or cell–based products such as Tregs
FDA	Considerations for the Design of Early-Phase Clinical Trials of Cellular and Gene Therapy Products	N	Jun 2015	Applies to the design of early-phase clinical trials for Treg products
FDA	Guidance for Industry: Preclinical Assessment of Investigational Cellular and Gene Therapy Products	N	Nov 2013	Applies to the design of pre-clinical studies using Treg products
FDA	Potency Tests for Cellular and Gene Therapy Products	N	Jan 2011	Applies to potency tests used for release of Tregs
FDA	Content and Review of Chemistry, Manufacturing, and Control (CMC) Information for Human Somatic Cell Therapy Investigational New Drug Applications (INDs)	N	Apr 2008	Recommends content to be provided in the IND submission if Tregs are human somatic cell therapy product
FDA	Eligibility Determination for Donors of Human Cells, Tissues, and Cellular and Tissue-Based Products	N	Aug 2007	Applies to screening of donors of human cells or cell–based products such as Tregs
FDA	Monoclonal Antibodies Used as Reagents in Drug Manufacturing	N	Mar 2001	Applies if monoclonal antibodies are used in the manufacturing process for Treg products
FDA	Guidance for Human Somatic Cell Therapy and Gene Therapy	N	Mar 1998	Applies if Tregs are somatic cells that have been manipulated or processed *ex vivo*

### Source of Tregs

Multiple sources of human Tregs have been explored clinically. Peripheral blood collected by apheresis or leukapheresis is the most accessible and often the only option for autologous therapy and is therefore the most used source of starting material to date. Human Tregs are relatively rare in PBMCs, comprising only 5-10% of peripheral CD4^+^ T cells in healthy adults, and this can be further reduced in patients with autoimmune disease (2, 9, 18, 19). This inherent variability in the percentage of circulating Tregs can be compounded in the variability in the amount of Tregs post isolation and increases the risk of manufacturing failures. Allogeneic Treg therapies manufactured from partially HLA-matched healthy donor-derived peripheral blood or umbilical cord blood (UCB) have been successfully (in Phase I studies) aimed at treating GvHD (12, 20). UCB is enriched in naive Tregs that possess inherent expansion potential and broad TCR repertoire. However, a single UCB unit contains significantly less Tregs than PBMCs (~5-7.5x10^6^), and Tregs derived from UCB often require multiple rounds of expansion (the implications of which are discussed later in this review) (12, 21).

Alternative sources of naive Tregs currently being explored for allogeneic products include pediatric thymuses, which are routinely removed during pediatric cardiac surgeries, and differentiation of iPSC or conventional T cells into Tregs. iPSC derived CD4^+^ Tregs are an emerging technology with the potential to address the current limitations of low yield related to ex vivo expansion, manufacturing variability and specificity. These cells have been shown to be as functional and suppressive as natural (CD4^+^CD25^+^CD127^-^) Tregs both ex vivo and in animal models of GvHD and can be concomitantly engineered during expansion to express CAR (22). iPSC derived Tregs offer a potentially renewable and scalable source of Tregs, however, as this class of cells are only being assessed at the preclinical stage, they will not be further discussed in this review. Using the thymus as the source of Tregs is advantageous due to the abundance of available Tregs. On average, 1-2% of thymocytes are CD4^+^CD25^+^ which translates to ~5x10^8^ cells. This is roughly 100x more than what is obtained from a UCB unit, and more than the number in the peripheral blood of an adult. The lack of activated conventional T cells in the thymus means that CD25^+^ exclusively marks CD4^+^ Tregs, which can facilitate isolation of a pure population of Tregs. The feasibility of manufacturing clinical material using this approach is currently being explored in a Phase I/II clinical trial assessing the ability of Tregs to prevent rejection after pediatric heart transplant (NCT04924491) (23) (24) (25). *In vitro* re-programming of conventional T cells into Tregs has been achieved through ectopic expression of Foxp3 (26, 27) as well as through culture with IL-2, rapamycin and TGF-B and was shown to be safe (NCT01634217) (13). Although feasible for clinical manufacturing, re-programming approaches require additional reagents as well as testing to prove the stability of the product, increasing the complexity of both manufacturing and release testing.

Human cells or tissues intended for implantation, transplantation, infusion or transfer into a human recipient are regulated as human cells, tissues and cellular and tissue-based products (HCT/Ps) under 21 CFR parts 1270 and 1271. These regulations require establishment registration, screening and testing of donors to reduce transmission of infectious diseases and also establish current good tissue practices (cGTP). Sponsors should think carefully about both the manufacturing and regulatory implications when choosing their starting material. While the manufacturing of allogeneic therapies is typically more reproducible than that of autologous therapies, the regulatory requirements for allogeneic products are more extensive than those for autologous products in order to assure patient safety. If multiple donors are being used, special attention must be paid to possible cell interactions that could result in unexpected immune responses or alter the performance of the cells.

### Treg isolation

Like conventional T cells, Tregs are functionally and phenotypically diverse and can be divided into subsets based on cell surface marker expression. Identifying whether particular subsets are more attractive therapeutically or whether the appropriateness of different subsets varies by application (i.e., GvHD vs, autoimmune disease) is essential to the field but at this point remains unknown. A detailed discussion and comparison of the different Treg subpopulations is beyond the scope of this review and is discussed in detail elsewhere (28, 29).

Due to their low frequency in all starting materials, Tregs must be purified prior to expansion by either magnetic-activated cell sorting (MACS) or fluorescent-activated cell sorting (FACS). As Tregs do not express any unique cell surface markers, there is considerable variation in the methods used to isolate Tregs for clinical use, with the only commonality between all methods being the use of CD25 as a positive selection parameter. Both MACS and FACS require specialized equipment and reagents, highly trained personnel and are labor intensive process steps that take several hours per sample (~6 hrs for MACS, >12 for FACS).

Magnetic sorting is performed with the CliniMACS Plus (Miltenyi Biotec), and Tregs can be isolated using a one- or two-step procedure. In one-step isolation protocols, CD25^+^ T cells are positively selected, whereas in two-step protocols, CD8^+^ and/or CD19^+^ are first positively depleted, then CD25^+^ cells are positively selected, yielding a CD4^+^CD25^+^ isolated product. The use of a one- vs. two-step protocol is largely determined based on the starting material. In allogeneic trials using UCB, isolation is typically performed with a one-step procedure as UCB cells express high levels of CD25 (CD25^high^), and the risk of CD25^+^ contaminating effector cells in this starting material is low. Conversely, two-step processes are common when peripheral blood is being used as the starting material. Post-isolation purity of the one-step process is typically low (<70%, median), whereas the two-step process yields significantly greater purity (>90%, median) (30). Isolation is a balance between selection of a sufficiently pure population and avoidance of relevant cell exclusion. Purity therefore comes at the cost of post isolation yield, regardless of the method or type of product.

Magnetic sorting is limited by the number of surface markers that can be used and is only capable of binary sorting based on the presence or absence of the selected markers. FACS is not only capable of using multiple markers but also allows for precise isolation of specific cell populations such as CD4^+^CD25^high^CD127^lo/-^ which have been shown to have a higher frequency of Foxp3 expression and greater potency *in vitro* (1). CD45RA expression identifies naïve Tregs and is another marker that is being included in FACS based isolation protocols. CD4^+^CD25^+^CD127^lo^ CD45RA^+^ Tregs isolated have been shown to have an epigenetically stable Foxp3 locus (TSDR demethylation), enhanced suppressive ability and reduced Th17 plasticity *in vitro* compared to CD4^+^CD25^+^CD127^lo^ CD45RA- Tregs (31–33). This approach was successfully used to isolate and expand Tregs in the TRIBUTE study (NCT03185000) for the treatment of inflammatory bowel disease (33). The post-isolation purity of FACS isolated Tregs can be as high as 98%, however cell recovery is often modest (<80% of input) due to loss of cells during the pre-enrichment and staining steps and the long processing time. Based on the indication and route of administration, a balance must be achieved between yield and purity. The lengthy processing time of FACS also limits the number of cells that can be processed via cell sorting, hampering the number of Tregs that can be isolated for expansion. In an effort to increase the number of cells that can be sorted using FACS, some groups perform magnetic sorting of CD4^+^ cells followed by FACS. This manufacturing process was found to yield a product with >95% Foxp3^+^ cells (34, 35). There are currently two 21 CFR part 11 compliant cell sorters used for cell sorting of adoptive cell therapies that can be qualified for use in a GMP environment; Miltenyi Biotec’s Tyto and Sony Biotechnology’s CGX10. The Tyto is a closed, cartridge style benchtop cell sorter equipped with three lasers, allowing for up to 10-parameter cell sorting. Sony Biotechnology’s fully closed CGX10 cell isolation system is equipped with four lasers and replaces the FX500, which utilized exchangeable fluidics and was housed in a Class II BSC to enable qualification for GMP use. The creation of flow cytometers that can be qualified for cell sorting has enabled the use of FACS in the manufacture of clinical products. This highlights the need for dedicated and expert personnel to maintain and operate these specialized instruments.

Regardless of the isolation approach used to purify Tregs, antibodies are the critical raw material in this phase of manufacturing. cGMP compliant MACS antibodies are available from Miltenyi Biotec for all of the routinely used isolation markers (CD4, CD8, CD19, and CD25). As these antibodies are produced by a single supplier, all cell therapy manufactures requiring these antibodies acquire them from the same source. This can result in high costs, long lead times and potential manufacturing delays if supply chain issues arise. While there are numerous suppliers of FACS antibodies, cGMP-compliant antibody options (clone and fluorochrome combinations) are limited. Care should be taken early in the planning of clinical manufacturing, as preclinical non-GMP reagents may never translate to the GMP environment, resulting in different processes. Should sponsors need to use an antibody that is not available in GMP format, the cost to create a controlled, cGMP-compliant antibody is > $1 million USD. As the antibodies are critical to the manufacturing process, lot to lot variability must be controlled and in later phases of clinical development, these critical raw materials will need to be tested and released to ensure control is maintained.

### *Ex vivo* expansion

Tregs are similar to conventional T cells in that they both requiring TCR and CD28 co-stimulatory signals for maximum expansion but are also highly dependent on IL-2 for survival, expansion and stability (9). Tregs are expanded using protocols similar to those used for conventional T cells; in culture bags or G-Rex bioreactors for 14–36 days, with CD3/CD28 stimulation and supplemented with IL-2 (9, 36). Anti-CD3/anti-CD28 coated microbeads and artificial APCs (aAPC) are the most widely used approaches for stimulating Treg expansion. However, cGMP compliant alternatives such as soluble CD3/CD28 antibody complexes have been developed (ImmunoCult Human CD3/CD28 T cell activator, STEMCELL and TransAct, Miltenyi) and are gaining popularity as they provide similar levels of activation, and are easily removed from culture media while minimizing cell loss. To reduce the risk of contaminating effector T cells, some groups add rapamycin, an approved mTOR inhibitor, to the expansion media to exploit the differential role of mTORC1 and mTORC2 in Tregs and effector T cells (Teff), resulting in the selective suppression of Teff growth due to the Foxp3 induced expression of Pim2, a serine/threonine kinase that confers rapamycin resistance (37, 38) (37–39). Although the addition of rapamycin improves purity, it reduces Treg expansion ~ 10 fold, necessitating longer ex vivo culturing times (11, 40). The addition of rapamycin is predominantly used when Tregs are isolated by CD4^+^CD25^+^ MACS to improve the purity of the final product. Expansion methods vary greatly between protocols with regards to culture media, additives, duration of culture and the type, timing and frequency of antigen stimulation, making it difficult to compare results across different products (11).

The use of autologous cells poses multiple challenges when it comes to expansion. First and foremost, in the reproducibility of the manufacturing process and the ability to produce an adequate number of cells. Tregs used in autologous therapies are isolated from the patient, so not only can the cells be dysfunctional, but there can be substantial variability in the extent of dysfunction between patients. This leads to heterogeneity during the expansion phase, both in the Tregs ability to expand and the phenotype of the final product (increased interferon production compared to Tregs from healthy donors) (36). Multiple trials have documented the failure of patient samples to meet the minimum cell number required for infusion (12, 20, 41). Due to the manufacturing challenges of autologous products, it is not uncommon for manufacturing feasibility to be included as a clinical endpoint of the trial. These issues underscore the need for a better understanding of starting material and their impact on Treg manufacturing. Through a deeper understanding, potential failures could be identified and manufacturing criteria adjusted to reduce the risk of failures.

Manufacturing of allogeneic Tregs from healthy donors is substantially more reproducible, although variability does exist. UCB is a common starting material for allogeneic therapies but due to the smaller number of Tregs in UCB, multiple rounds of expansion are necessary to achieve dose (12, 13), extending the cGMP manufacturing time and increasing the amount of reagents needed to manufacture, resulting in drastically higher manufacturing costs, diversion to a. While longer clinical expansion protocols have successfully manufactured Tregs with >75% CD4^+^CD25^+^Foxp3^+^ (38), prolonged expansion can have negative functional impact, such as diversion to a Th2 (IL-4 producing) Treg functional state (42). Thymic Tregs although naive, do not proliferate as effectively as UCB or blood Tregs during *ex vivo* expansion and are not widely implemented (19). However, recent advances in clinical grade expansion protocols for thymic Tregs have demonstrated the ability to produce highly pure, functional T-regs in 10–23 days (43). Moreover, the ability to cryopreserve and recover cells with >95% viability and 80% Foxp3+ post freeze-thaw further highlights the potential of this material for use in clinical manufacturing, making it likely to become a more prominent starting material in the future.

Ex vivo expansion protocols often require a large number of single use reagents and consumables, such as specialized media, cytokines (IL-2), antigen stimulation reagents (CD3/CD28 beads, artificial APCs), drugs (rapamycin), culture bags or bioreactors and serum. These raw materials and consumables must be released by the manufacturer for use. Often, for early phase raw material release can rely on the certificate of analysis presented by the manufacturer of the raw material. However, in situations where cGMP-compliant material may not be available, a risk assessment should be undertaken as to the impact of using research grade material in the manufacturing process of clinical material. This risk assessment, inclusive of an evaluation of controls that may be in place (such as in-process and/or release testing), may necessitate developing and conducting tests of the raw material itself. The test(s) should be conducted on the incoming raw material, upon receipt, for the release of the raw material for use in manufacturing by the product developer. Additionally, single-source reagents often pose a risk to the supply chain. In these situations, it is advisable to understand and monitor raw material and consumables lead time to receiving the material once an order is placed. A thorough understanding of material supply chain will allow for the ordering of safety stocks to mitigate supply chain associated risks to manufacturing campaigns.

In addition, the reagents used for Treg manufacturing can often introduce additional complexity. For example, Treg culture media is typically supplemented with 5-10% human AB serum to provide critical growth factors to cells. Not only is the serum costly, but it necessitates additional testing (both prior to use and during release testing) creating additional quality and compliance burden. Due to the variability of serum, serum lots must be qualified before release for use to confirm they support cell growth. Whenever a new lot of serum is to be used, an assessment of old and new lots using a reference material must be performed to confirm that expansion is similar. Serum-free media such as OpTmizer (ThermoFisher) and ImmunoCult-XF (StemCell) that contain serum replacement supplements are being used in the manufacture of other T cell therapies and may become more common in Treg manufacturing if it can be demonstrated that they enable similar levels of expansion as serum containing media.

The widespread implementation of cell-based therapies is limited by the complexity and scalability of the manufacturing process and the lack of a reliable manufacturing platform that ensures consistent production of clinical grade material at the required therapeutic doses. A variety of culture systems are currently used for the manufacture of cell-based therapies, and these are reviewed in detail elsewhere (44). Cell culture bags, G-Rex flasks and stirred flasks that are manufactured with gas permeable polymers or membranes are the simplest, least expensive and most widely used systems to date for manufacturing Treg therapies. These bags and flasks can have multiple aseptic ports for media input/output, sampling and harvest, but require additional equipment and manual processing at all steps. G-Rex flasks are among the most popular of these expansion systems due to their affordability and scalability.

Bioreactors (rocking motion (WAVE), stirred tank and hollow fiber (Quantum) are more mechanized culture systems that allow certain steps to be completed in a closed aseptic model, lowering the risk of operator-based errors during manufacturing. They can be equipped with pH and dissolved oxygen probes to provide real-time control of these variables. Currently, bioreactors are not commonly used in the manufacture of Tregs. Semi-automated and automated systems like the CliniMACS Prodigy and Lonza Cocoon require minimal human intervention. The CliniMACS Prodigy is a single closed system instrument capable of magnetic separation, cell expansion with automated media exchange and formulation. While not known to be capable of cell separation or formulation, the Cocoon supports cell transfection, transduction and expansion and can link several systems together in order to perform parallel large-scale cell expansion with full electronic control over the process in a single network, features especially beneficial for the commercial manufacturing of off-the-shelf allogeneic cell products (44). These cGMP-compliant systems reduce infrastructure requirements at the manufacturing facility however, they are costly and at the present moment, not more efficient in terms of time and yield than less advanced methods.

### Formulation

Upon completion of ex vivo expansion, cells must be formulated and filled for administration to patients. The final formulation of a drug product is dictated by indication, route of administration, storage requirements and shelf-life stability need. A key decision at this stage of Treg manufacturing is whether to cryopreserve the cells or administer them fresh. Cryopreservation is typically achieved through the use of cryopreservation solutions containing dimethyl sulfoxide (DMSO) and USP grade components, such as CryoStor CS10 (StemCell). Cell concentration is based on the intended clinical dose (cells/kg) and as such there is significant variability between trials as doses range from 1–100 x10^6^. Once formulated, cells are cryopreserved using controlled-rate freezers with validated freezing protocols, followed by long term storage in liquid nitrogen. This facilitates large-scale production at centralized manufacturing facilities, enables long-term storage and reduces logistical hurdles by allowing for flexibility around the time of infusion to accommodate changes in patient health, while also affording more time for release testing. Due to these benefits, cryopreservation has been widely implemented in the manufacture of both autologous and allogeneic cell-based therapies such as TIL and CAR-T. However, DMSO itself is a known inhibitor of viability and cryopreservation of PBMCs and purified Tregs cells has been associated with loss in cell yield, decreased viability as well as impaired cytokine production and suppressive capacity and reduced surface marker expression (FoxP3) that essential for proper Treg function (45–48). These effects can all dramatically affect the clinical safety and efficacy of this therapy (49, 50). Conversely, others have shown Tregs can be successfully frozen and thawed without compromising their phenotype (51–54). These discrepancies are likely due to the variations in the manufacturing process (starting material, method of expansion, purity of the final product) as well as differences in cryopreservation protocols (cryopreservation agent and % DMSO, cell concentration and freezing protocol), as there is currently no standard protocol. These mixed reports have led to uncertainty around the feasibility of cryopreserving Tregs, and as a result, clinical trials have been a mix of cryopreserved and freshly administered Tregs, with the majority using administering fresh products. Novel cryopreservation agents with potentially less deleterious effects on cell viability are currently being explored preclinically (55, 56). Development of cryopreservation media and standardized methods for Treg therapy should leverage the findings and progress being made in conventional T cell-based therapy, which is more clinically advanced.

## Product testing and release

The FDA mandates that cell-based therapy products demonstrate identity, purity, safety, potency (strength) and quality prior to administration to patients (**Table 3**). Product release testing must therefore address all of these features. While sterility and identity are relatively straightforward tests performed using standard analytical methods, demonstration of Treg purity and potency are more complex. Foxp3 expression is a good surrogate for assessing purity and the most routinely used measure of purity, however based on current isolation and expansion techniques, it is unlikely that 100% of cells will be Foxp3^+^. Moreover, Foxp3 Tregs are phenotypically and functionally heterogeneous and Foxp3 expression can be induced by TCR stimulation of T effector cells which do not possess suppressive activity (57) As such, Foxp3 expression is necessary, but not sufficient to identify functional human Tregs, requiring the co-expression of additional markers such as CD3 CD4,CD25, CD127, CD45RA and absence of contaminating cell markers (CD8^+^, CD56^+^) to accurately identify Tregs and assess purity (58). Acceptance criteria must therefore determine acceptable levels of product related impurities. The level of acceptable impurities will vary based on the starting material used and the therapeutic indication. For example, therapies for the treatment of Type I diabetes (a reasonably well controlled autoimmune disease), often have higher acceptable proportion of Foxp3^-^ cells than therapies for severe disease like GvHD where high numbers of conventional T cells underlie the diseases.

**Table 3:** Common release tests for Treg therapies.

Category	Test	Analytical method	Material for testing
Quality	Appearance	Visual	DP
	Cell Viability	Cell counter (Trypan Blue)	DS, DP
	Total Viable Cell Count	Cell Counter	DS, DP
Identity	Treg Phenotyping	Flow Cytometry (CD3+CD4+CD25+ AND absence of CD8+ and NK cells (CD3+CD56+)). Antibodies used for identity depend on how Tregs were isolated and may include additional markers then those listed here.	DS, DP
Purity	Residual CD3/CD28 Beads	Flow Cytometry	DS
Safety	Endotoxin	USP <85>	DP
	Mycoplasma	USP <63> or validated qPCR	DS/Cell Lysate
	Sterility	USP<71>, Gram stain or BacT ALERT	DS, DP
Strength (Potency)	Functional Potency	Proliferation of Effector T Cells (early-phase trials) OR Quantification of suppressive cytokines & inhibitory receptor expression known to correlate with function (late-stage/commercial)	DP

Our understanding of Treg function lags behind that of conventional T cells. While multiple mechanisms have been implicated in Treg function, a clear understanding of how suppression occurs *in vivo*, and which *in vitro* surrogates correlate with efficacy remains to be elucidated. Suppression assays that assess the ability of Tregs to inhibit proliferation of effector T (Teff) cells are the most commonly used method for quantifying functional potency. In these assays, Teff cell proliferation is quantified by [^3^H] Thymidine incorporation or CFSE dilution following TCR stimulation (i.e., CD3/CD28 beads) and co-culture at different Treg: Teff ratios (59). Surrogates of Treg potency include the secretion of suppressive cytokines such as IL-10, IL-35 and TGF-B (contact independent inhibition), and expression of inhibitory molecules such as CTLA-4, LAG3, CD39, ICOS (contact dependent inhibition) (28, 60, 61). These surrogates can be correlated with *in vitro* bioactivity/function and utilized for late-stage and commercial potency lot release and stability, which are likely to be more easily validated than a bioactivity/functional *in vitro* assay like inhibition of cell proliferation.

## Stability and logistics

Stability of Tregs, similar to other cell therapies, is controlled by whether the product is administered fresh or frozen. Cryopreserved Tregs will undergo challenges to ensure that controlled freeze and thaw as well as formulation that is supportive of the storage condition maintains the viability requirements of the final product. Freshly administered product, on the other hand will have a very short shelf life and the location of the clinical site often dictates where manufacturing occurs. For freshly administered drug products, decentralized manufacturing is often explored to ensure timely delivery of the final product to the patient.

In addition to the general stability concerns for cell therapies, Treg therapies have a unique stability risk post infusion due to their inherent plasticity - the possibility of expanded Tregs reverting to effector T cells (T_H_1, T_H_2 and T_H_17) through the loss of Foxp3 expression. Tregs have remarkable phenotypic plasticity, with the ability to acquire different transcriptional programs in response to the environment, leading to the generation of functionally distinct subsets (62). The basis of this plasticity is their ability to express different master regulatory transcription factors, and it poses a significant quality risk as the infused product has the potential to become unstable and develop unwanted activities (i.e., effector functions) that can cause serious harm (63, 64). Overexpression of Foxp3 has been proposed as a strategy to stabilize Tregs *in vivo* (5). Peripheral Tregs have been reported to be less stable than thymic Tregs under lymphopenic conditions, indicating thymic Tregs may represent a better population for ACT (63). With the majority of Treg therapies derived from peripheral blood starting material, the ability to monitor and accurately characterize the phenotype and function of infused cells is essential to ensure product safety. Currently, the best markers for monitoring Treg stability are demethylation of the Treg specific demethylated region (TSDR) which is needed for sustained Foxp3 expression by dividing Tregs (65) as well as expression of Foxp3, CTLA-4 and PD-1, proteins constitutively expressed in functional Tregs, and expression of alternate lineage transcription factors such as T-bet, GATA3, Bcl-6 and RORγt, required for T_H1_, T_H2_, T_FH_ and T_H17_ (4, 5). Several of the transcription factors driving Foxp3 expression (STAT5, Ets-1 and Foxp3 itself) preferentially bind to hypomethylated regions in the Foxp3 gene, linking demethylation with stabilized transcription as a key determinant of Treg stability. Understanding why cells lose their “Treg” state and preventing dedifferentiation *in vivo* are critical to improving both the safety and efficacy of Treg therapy.

## Additional considerations

Dosing and persistence: Optimal Treg dose for clinical application has yet to be determined. While some products have been shown to persist and retain their CD25^+^Foxp3^+^ phenotype in the circulation for up to 1 year, the same study showed rapid decline in the percentage of infused Tregs, with most being undetectable within 90 days of administration.

Comparability: Given the discussed variability in the starting material and potential association with variability in the final product yield, assessing comparability when there has been change to the manufacturing process will pose unique challenges to a Treg program. In addition, purity and potency are inherently challenging attributes to evaluate in a Treg product. Together, yield for dosing, purity for safety, and potency for strength will be deemed critical quality attributes of any clinical product. The challenge in developing a controlled manufacturing process that will reduce the variability in these three attributes to generate a consistent Treg product, will be amplified if a comparability assessment needs to be made. Establishing equivalence, when the attribute displays a high level of variability, may prove to be a time consuming, costly, and challenging task.

## Conclusions

Tregs are widely regarded as the primary cells involved in the persistence of immune tolerance. They exhibit broad bystander suppression and can also mediate tolerance, amplifying the impact of the cells and resulting in robust and durable efficacy. Over the past decade, cell therapies have seen exponential clinical and market growth, and Treg therapies for the treatment of autoimmune diseases, inflammation and graft/organ transplantation are no exception. As living drugs, all cell therapies face a battery of manufacturing, analytical and regulatory complexities and hurdles. In addition to these field wide complexities, Treg therapies are faced with the unique challenges of how to define purity, assess *in vivo* potency *in vitro* and monitor the stability of infused cells. Treg therapies address therapeutic areas with few other options for therapy or cure, as such the need to invest in these therapies to truly understand the capabilities and realize their promise is great.
